# Combined *GSTM1* and *GSTT1* null genotypes are strong risk factors for atherogenesis in a Serbian population

**DOI:** 10.1590/1678-4685-GMB-2017-0034

**Published:** 2018-03-26

**Authors:** Ivana Grubisa, Petar Otasevic, Nada Vucinic, Biljana Milicic, Tanja Jozic, Slobodan Krstic, Jelena Milasin

**Affiliations:** 1 Department of Human Genetics and Prenatal Diagnostics, Zvezdara, University Medical Center, University of Belgrade, Belgrade, Serbia; 2 Dedinje Cardiovascular Institute Belgrade, Serbia; 3 Department of Statistics, School of Dental Medicine, University of Belgrade, Belgrade, Serbia; 4 Clinic of Cardiology, Clinical Center of Serbia, Belgrade, Serbia; 5 Clinic for Emergency Surgery, Emergency Center, Clinical Center of Serbia, Belgrade, Serbia; 6 Faculty of Medicine, University of Belgrade, Belgrade, Serbia; 7 Department of Human Genetics, School of Dental Medicine, University of Belgrade, Belgrade, Serbia

**Keywords:** atherosclerosis, gene polymorphisms, glutathione S-transferases, oxidative stress

## Abstract

Oxidative stress (OS) plays an important role in atherogenesis and since glutathione S-transferases (GSTs) provide protection against OS, we have tested the hypothesis that deletion polymorphisms in two GSTs (*GSTM1* and *GSTT1*) may affect the risk of developing atherosclerosis. A total of 382 individuals (200 patients with atherosclerosis and 182 healthy controls) were included in this association study. Genomic DNA was isolated from peripheral blood cells or from buccal epithelial cells and genotyping was performed using multiplex-PCR or real-time PCR methods. *GSTM1* null genotype was significantly more frequent in atherosclerotic patients than in controls (52.0% *vs* 34.1%) and individuals with the *GSTM1* null genotype had an approximately 2-fold increase in atherosclerosis risk (OR: 2.1, 95%CI=1.39-3.17, P=0.0004). *GSTT1* null genotype alone did not show a statistically significant effect on atherosclerosis risk modulation, but the association approached significance (OR: 1.57, 95%CI=0.94-2.64, *P*=0.08). The combined analysis showed that the presence of both genes had a protective effect against atherosclerosis (OR=0.55, 95%CI=0.37-0.83, *P*=0.005) while double null genotypes led to a robust atherosclerosis risk increase (OR: 8.14, 95%CI= 2.41-27.51, P < 0.0001). This study demonstrated that the *GSTM1* null and combined *GSTM1/GSTT1* null genotypes are susceptibility factors for development of atherosclerosis in a Serbian population.

## Introduction

Substantial data indicate that oxidative stress (OS) is involved in the development of atherosclerosis. Atherogenesis, one of the main risk factors for cardiovascular diseases (CVD), is initiated by oxidation of the low-density lipoprotein (LDL) and by impairment of oxidative stress-antioxidant balance. Conventional risk factors for CVD, including diabetes mellitus, hypercholesterolemia or dyslipidemia, hypertension and smoking, are generally related to OS. Patients with CVD commonly have at least one identifiable risk factor but many ischemic events occur in the absence of risk factors (Futterman and Lemberg, 1998). For this reason, there has been a deep interest in finding additional markers of oxidative stress, including gene polymorphisms, which might be used as predictors of disease risk.

Reactive oxygen species (ROS) produced by endothelial cells, smooth muscle cells (SMCs) and macrophages oxidize LDL in the subendothelial space, at the sites of endothelial damage, initiating events that culminate in the formation of atheroma. Some antioxidant enzymes such as superoxide dismutase and catalase directly eliminate ROS, while gluthatione S- transferases (GSTs) detoxify secondary cytotoxic metabolites of ROS. As phase II biotransformation enzymes, GSTs protect cellular macromolecules by catalyzing the conjugation of reduced glutathione (GSH) to exogenous and endogenous electrophilic compounds, which are, respectively, the results of either xenobiotic breakdown, or oxidative damage to lipids and DNA (Douglas, 1987; Berhane *et al.*, 1994; Hayes and McLellan, 1999). Specific intracellular localization of the members of this GST superfamily has facilitated their categorization into three distinct groups: cytosolic, mitochondrial and microsomal GSTs. Cytosolic GSTs represent the largest family, and genes encoding these soluble isoforms are spread throughout the genome (Hayes and Strange, 2000). Polymorphisms in GST genes affect the activity of encoded enzymes and consequently have functional effects on redox regulation. A variety of polymorphisms have been described in the GSTs gene superfamily, but major attention has been focused on complete deletion polymorphism in mu (GSTM) and theta (GSTT) subfamilies since they abolish enzymatic activity. Polymorphic variants in the *GSTM1* (GSTM1*0) and *GSTT1* (GSTT1*0) genes produce either a functional proteins, i.e. non-null phenotypes (non-deletion alleles or heterozygous deletion) or result in the complete absence of enzymes, i.e. null phenotypes (homozygous deletion or null genotype) (Eaton and Bammler, 1999).

Many conditions have been associated with the deletion polymorphisms in *GSTM* and G*STT* genes, including asthma (Lima *et al.*, 2010), male infertility (Dordevic *et al.*, 2010), end-stage renal disease (Suvakov *et al.*, 2013), leukemia (Souza *et al.*, 2008; Nasr *et al.*, 2015) and lung cancer (Sharma *et al.*, 2015; Yang *et al.*, 2015), among others.

So far, controversial results have been reported about the importance of *GSTM1* and *GSTT1* null genotypes in atherogenesis in different human populations (Türkanoglu *et al.*, 2010; Ramprasath *et al.*, 2011; Nørskov *et al.*, 2011; Santl Letonja *et al.*, 2012; Cora *et al.*, 2013; Yeh *et al.*, 2013; Mir *et al.*, 2016). This has prompted us to study the potential role of deletion polymorphisms in *GSTM1* and *GSTT1* in predicting the susceptibility to atherosclerosis in Serbian patients.

## Material and Methods

### Study population

A total of 382 individuals (200 patients and 182 controls) participated in this case-control study. All participants were of Serbian ethnicity and unrelated. Patients presented clinical manifestations of atherosclerosis and had been treated at Dedinje Cardiovascular Institute (DCI) or Zvezdara University Medical Center (ZUMC), Belgrade, Serbia, from 2010 to 2011.

Diagnosis of atherosclerosis was based on medical history including: presence of coronary heart disease (CHD), cerebrovascular diseases (CVD), peripheral arterial disease (PAD) and type 2 diabetes mellitus (T2DM). CHD in patients with previous myocardial infarction and stable angina pectoris was estimated by coronarography, whilst carotid atherosclerosis was estimated by high-resolution B-mode ultrasonography HDI, ATL 3500, or according to data related to carotid artery surgery. Cerebral ischemia was diagnosed according to symptoms including amaurosis fugax, transient ischemic attack and stroke, and PAD was diagnosed by ankle/brachial index (<0.90) or according to previous data about lower extremity arterial surgery. The determination of T2DM was based on values of glycemia (2 readings of glycemia in 2 consecutive days): fasting plasma glucose concentration ≥7.0 mmol/L (126 mg/dL) or glycemia in any random blood sample (regardless of meals) ≥ 11.1 mmol/L (200 mg/dL) with the presence of typical diabetes symptoms (polyuria, polydipsia, weight loss) or based on the value of glycemia during an oral glucose tolerance test (OGTT): plasma glucose concentration during an OGTT in the 120^th^ minute ≥ 11.1 mmol/L (200 mg/dL). Blood pressure measurements were done according to recommendation of the Seventh Report of the Joint National Committee on Prevention, Detection, Evaluation, and Treatment of High Blood Pressure (JNC7) (Chobanian *et al.*, 2003): participants were considered hypertensive if their average (the mean of at least two measurements) systolic blood pressure (SBP) was 140 mmHg or higher and/or their average (the mean of at least two measurements) diastolic blood pressure (DBP) was 90 mmHg or higher (and/or the person used antihypertensive medications). Hyperlipidemia was diagnosed if a person’s complete lipid profile had total cholesterol level ≥6.20 mmol/L, triglyceride level ≥2.3 mmol/L, low density lipoprotein level ≥ 4.90 mmol/L and high density lipoprotein values ≤1.00 mmol/L or if a person used lipid-lowering medications. Participants were classified as smokers if they were former or current smokers, irrespective of the number of cigarettes smoked daily. Overweight or obesity was defined as body mass index (BMI) ≥ 25 kg/m^2^. Exclusion criteria were malignancy and any chronic inflammatory disease.

The control group was composed of 182 individuals who showed no evidence of CHD, CVD, PAD, T2DM, hypertension, malignancy and chronic inflammatory diseases based on laboratory and clinical checkup at ZUMC’s clinics. The percentage of female *vs*. male participants in the control group corresponded to the percentage in the case group. Similarly, the age range and mean were approximately the same in the two groups.

All procedures were done in accordance with Helsinki Declaration of 1975 and the study protocol was approved by the ethical Committee of Zvezdara University Medical Center. All study participants signed the informed consent.

### Genetic analysis

Genomic DNA was isolated from peripheral blood or from buccal swabs by DNeasy Blood & Tissue Kit (Qiagen) according to the manufacturer’s recommendations. Isolated DNA was stored at 4 °C until further analysis.

For simultaneous detection of *GSTM1* and *GSTT1* genotypes a multiplex polymerase chain reaction (multiplex PCR) and real-time PCR were performed with primers described by Voso and coworkers (Voso *et al.*, 2002). As an internal amplification control, primers for *β*-globin gene were used to exclude false negative results.

PCR mixture for multiplex PCR (total volume 50 μL) contained 2X Multiplex PCR Master Mix (2X-concentrated solution containing HotStart Taq DNA polymerase, reaction buffer, MgCl_2_ and dNTP (Qiagen), 0.5 μM of each primer (Metabion) and 0.2 μg of genomic DNA. Amplification products from this reaction were separated on 3% agarose gels, stained with ethidium bromide (0.5 μ/mL) and visualized under UV light for determination of genotypes. Subjects with *GSTM1* and *GSTT1* null genotypes did not show amplification of fragments corresponding to 215 bp and 480 bp, respectively. An amplified *β*-globin gene fragment (110 bp) was observed in every PCR reaction as an indicator of a successful reaction.

Instead of randomly re-genotyping 10% of samples by the same multiplex PCR procedure to confirm the genotypes, 50 samples were additionally genotyped by real-time PCR and melting curve analysis. No discrepancies between genotypes determined in duplicate were found. Reaction mixes for real-time PCR were prepared according to the manufacturer’s recommendations (for a total volume of 25 μL): 2X Maxim SYBR Green/ROX qPCR Master Mix (Fermentas Life Sciences), 0.35 μM of each primer (Metabion), <500 ng template DNA and nuclease-free water to complete 25 μl.

### Statistical analysis

Chi-square (X^2^) and Student’s *t*-test were used to compare demographics and clinical characteristics between case and control groups. Allele and genotype frequencies were estimated by allele counting method. Possible differences in allele and genotype frequencies between cases and controls were determined by Pearson’s X^2^ test and Fisher’s exact probability test. The risk of disease of examined genetic polymorphisms was assessed by calculating odds ratios (OR) and their 95% confidence intervals (CI). A *P-*value < 0.05 was considered significant. Statistical analysis was carried out using SPSS software (version 17.0). Enrollment of 200 atherosclerotic patients and 182 healthy controls achieved a 97.2% power to detect a significant difference in development of atherosclerosis between the observed groups, at a two-tailed significance level of 0.05 using chi square test. Also, this sample size achieved a 99.3% power when we observed the frequency of *GSTM1* and *GSTT1* double null genotypes.

## Results

The demographic and clinical characteristics of the studied groups are shown in Table 1. There was no significant difference between cases and controls in terms of age, sex, obesity, hyperlipidemia and smoking. In contrast, there were significantly more subjects with T2DM and hypertension among patients with atherosclerosis than among controls (*P* < 0.0001 for both variables).

**Table 1:** Demographic and clinical characteristic of atherosclerosis patients and controls.

Variables	Patients (N=200)	Controls (N=182)	*P*
Age (years)	60.3 ± 6.3	59.5 ± 3.4	0.12^a^
Gender, M/F N (%)	131/69 (65.5/34.5)	118/64 (64.8/35.3)	0.89^b^
Overweight or obese N (%)	112 (56.0)	91 (50.0)	0.2^b^
Diabetes Mellitus Type 2 N (%)	140 (70.0)	-	<**0.0001** ^b^
Hypertension N (%)	178 (89.0)	-	<**0.0001** ^b^
Hyperlipidemia N (%)	170 (85.0)	142 (78.0)	0.07^b^
Smoker (current or former) N (%)	98 (49.0)	78 (42.8)	0.27^b^

Data for age are presented as mean ± SD, N: number of subjects; *P*: probability; ^a^Student’s *t*-test; ^b^X^2^ test

A significant difference in *GSTM1* null genotype (phenotype) frequencies between cases and control patients was observed (52.0 versus 34.1%). No association was found between *GSTT1* null genotype (phenotype) and disease but the risk approached significance (*P*=0.08) (Table 2). *GSTM1/GSTT1* combined phenotype frequencies were significantly different between cases and controls for *GSTM1* non-null*/GSTT* non-null and *GSTM* null*/GSTT* null. The presence of both genes had a protective effect (OR=0.55, 95%CI=0.37-0.83, *P*=0.005) whilst individuals with double null genotypes had an approximate 8-fold increase of the risk for atherogenesis (OR=8.14, 95% CI=2.41-27.51, *P*<0.0001) (Table 3).

**Table 2:** *GSTM1* and *GSTT1* phenotype frequencies in patient and control groups.

Phenotypes	Patients/(200) N (%)	Controls (182) N (%)	OR	95%CI	*P*
**GSTM1**					
non-null (+/+, +/-)	96 (48)	120 (65.9)			
null (-/-)	104 (52)	62 (34.1)	2.1	1.39-3.17	**0.0004**
**GSTT1**					
non-null (+/+, +/-)	154(77)	153 (84.1)			
null (-/-)	46 (23)	29 (15.9)	1.57	0.94-2.64	0.08

N: number of subjects; OR: odds ratio; 95%CI: 95% confidence interval; *P*: probability

**Table 3:** Odds ratio analysis of combined *GSTM1/GSTT1* phenotypes in patients and controls.

*GSTM1/GSTT1* phenotypes	Patients (200) N (%)	Controls (182) N (%)	OR	95%CI	*P*
Non-null/Non-null	74 (37)	94 (51.7)	0.55	0.37-0.83	**0.005**
Non-null/Null	22 (11)	26 (14.3)	0.74	0.40-1.36	0.417
Null/Non-null	80 (40)	59 (32.4)	1.39	0.91-2.12	0.152
Null/Null	24 (12)	3 (1.6)	8.14	2.41-27.51	**0.0001**

N: Number of subjects; OR: odds ratio; 95%CI: 95% confidence interval; *P*: probability

## Discussion

It is well established that genetic factors play an important role in the pathogenesis of atherosclerosis and that genetically susceptible individuals are likely to develop the disease when exposed to endogenous and environmental risk factors, among others.

OS is the result of overproduction of reactive oxygen species and/or deficiency of antioxidant mechanisms and depends on the balance between generation of ROS and enzymatic or non-enzymatic systems of antioxidative protection. Several factors, such as hypercholesterolemia, hypertension, diabetes, obesity and aging are established risk factors for atherosclerosis-based cardiovascular diseases where OS is increased and antioxidant defenses are compromised.

GSTs, as antioxidant enzymes, represent the second line of defense, which neutralizes lipid peroxidation products (Sharma *et al.*, 2006). Given the high global prevalence of CVDs, the number of studies dealing with the effects of GST polymorphisms on atherosclerosis are actually quite limited. Not only are the studies insufficient in number, but also the results are diverse and sometimes contradictory, which is in part the consequence of ethnic and geographical specificities of the studied populations.

A recent analysis of functional genetic differences in GST enzymes among six human groups with different ethnic background has shown that ethnicity strongly affects the genetic variability of GST enzymes. These data emphasize that human populations have different structure of detoxification genes, suggesting that ethnic differences influence disease risk or response to drugs (Hiragi *et al.*, 2011; Polimanti *et al.*, 2013).

In four large studies and meta-analyses in Danes, copy number variation (CNV) in *GSTM1* and *GSTT1* was not associated with risk of any ischemic vascular event or with markers of inflammation (Nørskov *et al.*, 2011). A meta-analysis performed by Wang *et al.* (2010) suggested that the *GSTM1* null genotype may slightly increase the risk of coronary heart disease and that interaction between unfavorable *GSTs* genotypes may exist.

In a Turkish population, *GSTT1* and *GSTM1* null genotypes, together with hypertension, were seen to play a significant role in the pathogenesis of ischemic stroke (Türkanoglu *et al.*, 2010). It has also been reported that the *GSTT1* null genotype is associated with premature morbidity and mortality in individuals with T2DM (Doney *et al.*, 2005), and a very clear association has been shown between the *GSTT1* null genotype and combined *GSTM1* and *GSTT1* null genotypes, and complications related to diabetes such as atherosclerosis (Ramprasath *et al.*, 2011; Santl Letonja *et al.*, 2012).

In the present study, a significant difference in *GSTM1* genotype distribution was found between cases and controls and homozygous *GSTM1* deletion significantly increased the risk of clinical manifestations of atherosclerosis. This risk increase was even more pronounced in individuals with the deletion of both genes.

The level of expression of GSTs is a crucial factor in determining the sensitivity of cells to a broad spectrum of toxic chemicals. GSTs are highly expressed enzymes with a complex transcriptional and posttranscriptional regulation and with structurally very different inducers, many of them being substrates at the same time. Patients with genetic variations that result in the absence or very little corresponding enzyme, show more severe clinical as well as biochemical and cellular phenotypes than patients carrying variations that still produce some residual protein amounts and enzyme activity (Olsen *et al.*, 2013). GST induction is considered part of an adaptive response mechanism to chemical stress caused by electrophiles. Since GSTs production determines an individual’s ability to detoxify products of ROS, especially endogenous products of oxidative damage, double deletions of *GSTM1* and *GSTT1* resulting in lower levels of GSTs are likely to play an important role in the development of diseases related to oxidative stress, including atherosclerosis.

A major contribution of a single gene is very unlikely in multifactorial diseases such as atherosclerosis, where a large number of pathogenetic mechanisms lead to a clinical outcome (Dalepiane *et al.*, 2007). Thus effects of a loss-of-function polymorphism alone are probably not sufficient to influence the progression of atherosclerosis, but the accumulation of “unfavorable” oxidative stress-associated gene polymorphisms is likely associated with the evolution of its clinical manifestations (Katakami *et al.*, 2009).

From the present study, it seems that *GSTM1* null and combined *GSTM1*/*GSTT1* null phenotypes are strong risk factors for atherogenesis in the Serbian population. It must be emphasized however, that the statistical power to detect association may be limited due to the relatively small sample size of both atherosclerotic patients (200) and controls (182), leading to false positive results. Recruitment and survival bias cannot be ruled out either, in particular among controls who may develop atherosclerosis in the future (Guo *et al.*, 2013). A study on a larger group of atherosclerotic patients, with and without diabetes mellitus should also be considered in order to avoid this confounding element.

In conclusion, coupled deletions of *GSTM1* and *GSTT1* genes may play a significant role in the etiopathogenesis of atherosclerosis and may represent a useful marker in the prediction of disease susceptibility in Serbs.
